# Disseminating Ambient Assisted Living in Rural Areas

**DOI:** 10.3390/s140813496

**Published:** 2014-07-25

**Authors:** Gerhard Leitner, Alexander Felfernig, Anton J. Fercher, Martin Hitz

**Affiliations:** 1 Institute for Informatics Systems, Alpen Adria Universitaet Klagenfurt, Universitaetsstrasse 65-67, 9020 Klagenfurt, Austria; E-Mails: anton@isys.uni-klu.ac.at (A.J.F.); martin.hitz@aau.at (M.H.); 2 Institute for Softwaretechnology, Graz University of Technology, Inffeldgasse 16B, 8020 Graz, Austria; E-Mail: alexander.felfernig@ist.tugraz.at

**Keywords:** ambient assisted living, smart home, field study, real-world application, rural area

## Abstract

The smart home, ambient intelligence and ambient assisted living have been intensively researched for decades. Although rural areas are an important potential market, because they represent about 80% of the territory of the EU countries and around 125 million inhabitants, there is currently a lack of applicable AAL solutions. This paper discusses the theoretical foundations of AAL in rural areas. This discussion is underlined by the achievements of the empirical field study, Casa Vecchia, which has been carried out over a four-year period in a rural area in Austria. The major goal of Casa Vecchia was to evaluate the feasibility of a specific form of AAL for rural areas: bringing AAL technology to the homes of the elderly, rather than moving seniors to special-equipped care facilities. The Casa Vecchia project thoroughly investigated the possibilities, challenges and drawbacks of AAL related to this specific approach. The findings are promising and somewhat surprising and indicate that further technical, interactional and socio-psychological research is required to make AAL in rural areas reasonable in the future.

## 1. Introduction

Ambient intelligence (AmI), in the definition of [1], is “aiming at a proactive, but sensible support of people in their daily lives”. There are many possibilities imaginable where this kind of support would make sense, for example in working, leisure and living contexts. There is currently a new wave of interest in ambient assisted living (AAL), which can be seen as a specific type of AmI aimed at supporting the elderly in their living contexts. Meanwhile, a wide variety of AmI functionality is available that could be meaningfully applied in AAL. Such an application requires a stable, but flexible technical infrastructure upon which to build. In the private residential building sector, technology subsumed under the term smart home could serve as such an infrastructure. Given that the smart home has long been a matter of interest in industry, as well as in academia, it could be expected that the technical prerequisites for providing AAL should be easily fulfilled. However, although the smart home was introduced about half a century ago, a private smart home is still the exception, rather than the rule. High expectations had to be repeatedly revised downwards in the past [2,3], but the technological prerequisites are not the only uncertainties to be considered in order to transform AAL [4] from fiction to future.

The Casa Vecchia project, which is presented in this paper, follows new approaches, which make it different from the mainstream of AAL research. It is, to our knowledge, one of only a few projects that are able to integrate and adapt state-of-the-art AAL technology into the almost arbitrary real-world living environments of seniors. Within a four-year period, the challenges, threads and possibilities of AAL on a technical and socio-psychological level, were thoroughly investigated.

In this paper, the theoretical aspects of AAL are described and considered in the context of the empirical findings of Casa Vecchia, a longitudinal field study, which was carried out between spring, 2010, and spring, 2014, in a rural area of Austria. The remainder of this paper is structured as follows. In Section 2, an overview of related work is given, and the requirements derived from this work in order to achieve the goal of AAL in rural areas are enumerated. Section 3 contains a thorough description of the approaches followed in Casa Vecchia. Research results are presented with a focus on the technological aspects in Section 4. In the final Sections 5 and 6, the results and the lessons learned are discussed, and an outlook on future work is given.

## 2. Related Work

Since the introduction of the smart home in the 1970s, AmI around the turn of the millennium [1,5,6] and AAL following the middle of the 2000s [4,7,8], numerous research and development activities have been carried out under the umbrella term, smart environments. An extensive body of achievements consisting of theoretical knowledge, as well as a broad range of system architectures and prototypes are available. The approach followed and described in this paper was therefore not to reinvent any one of the multitude of existing wheels, but to build upon those achievements and to customize them to the specific needs of AAL in rural areas. The following sections give an overview on the relevant theoretical aspects and practical foundations that have to be taken into consideration to achieve this goal.

### 2.1. Relevance of Rural Areas

The reason for focusing Casa Vecchia on rural areas was that, despite their socio-political relevance, they are under-represented in AAL research. As an illustration, consider that 80% of the territory in the European union is rural and that it is occupied by 25% of the overall population, or 125 million people [9,10]. Given the current estimates of impending demographic changes, the demands involved in supporting the rapidly-growing group of elderly people will be a big challenge for society. Furthermore, because of the phenomenon of rural escape, the ratio of elderly to other age groups will be a specific challenge for rural areas. Adverse conditions prevailing in rural area, such as suboptimal infrastructure and long distances, put additional pressures on the situation. Moving the elderly into care facilities is one proposed solution to the problems of demographic change, despite the fact that the great majority of elderly do not want to leave their homes to which they are accustomed [11]. Moving rural inhabitants to nursing facilities would have specific side effects, because of the adverse conditions mentioned above: leaving a rural residence often also means losing one's social network. The approach followed in the Casa Vecchia project was therefore to investigate the possibilities of bringing AAL technology to the ancestral homes in which the seniors are currently living instead of moving them into centralized institutions. We have addressed the challenges, costs and benefits of this approach in order to examine whether this constitutes a reasonable alternative to care facilities.

### 2.2. Technical Foundations

Towards making this a reality, it was necessary to identify the state-of-the-art and to find an adequate technological basis to enable AAL at home. The majority of residences in rural areas are stand-alone family homes. This is also true for the region in which the Casa Vecchia project was initiated. Because of the real-world limitations of dealing with this type of housing, we started by reviewing the options available to consumers under the label smart home. Two major categories of systems are offered: wired systems, on the one hand, and wireless systems, on the other, both having their advantages and disadvantages. Wired systems require the installation of additional infrastructure (bus wires) and are, because of the related efforts and costs, not optimally suitable for retrofitting. In comparison, wireless systems are operated via radio control, do not require much additional infrastructure and are therefore more economic and easier to retrofit. Specifically, their low price has probably led to a wide variety of systems and components offered on the Internet, in hardware stores or at electronic retail stores. The majority of these systems represent islands of functionality with the biggest drawback being that, in contrast to wired systems, there are no commonly-accepted standards. A custom combination of components (because not every system offers the same spectrum of functions) is difficult or even impossible, because of the lack of interoperability. Given the requirement of Casa Vecchia to customize AAL technology into any living environment, this was a major issue. However, developments, such as service-oriented layered architectures (SOAs) and the open service gateway initiative (OSGi) (www.osgi.org) help to overcome this problem by enabling custom solutions. They have already been successfully applied in AAL and smart home projects (e.g., Amigo or Soprano), *cf.* [12–14]. On the basis of these architectures, it is possible to combine components from different manufacturers and to choose them based on functionality rather than on brand. As will be described in Section 3, one of the first and central tasks in Casa Vecchia was to build a custom platform that enabled the integration of components of three different smart home systems covering the required spectrum of functionality.

### 2.3. Economic Aspects

Choosing an adequate technology is not only a question of functionality and compatibility, but also a matter of cost. In the related literature, cost is frequently cited as a causal factor in the low rate of dissemination of smart homes. Development in rural areas is specifically challenged by the cost aspect, because the average income (and the portion of it that is disposable) is low compared to urban regions [15]. Therefore, the universal application of AAL technology to rural areas will only become viable when the market offers solutions that are economically accessible to the consumer. This section focuses on the direct economic demands on potential AAL users and intentionally leaves out the discussion of cost aspects on a societal level. A review of the literature shows that basic smart functionality can start at a price level as low as € 150 for do-it-yourself (DiY) solutions, but can also cost up to € 90,000 for full-fledged smart homes with outsourced professional maintenance [16,17]. An important way to keep prices under control is to carefully analyze the requirements [18] and to select only functions and components that are unconditionally necessary. Although this need is clear, it is closely linked to the technical limitation described in the previous section. Living conditions and needs are dynamic and change over time. Implementing an AAL system with a limited functional range only makes sense when it is ensured that the system allows reasonably easy extension and adaptation at a later point of time. Casa Vecchia therefore was conceived of as a practical and immediately practicable project that can overcome these technical and financial obstacles. A system fulfilling these requirements has to provide a stable technical basis in combination with high flexibility in order to accommodate both current and future needs. Moreover, the system should be affordable for people with average income and should be adaptable on demand. This meant that we had to develop a system that could expand over time as required, but that could begin with a very basic and inexpensive installation. The functional aspects of our solution to these challenges are described in the next section.

### 2.4. Functional Aspects

Having analyzed the technical requirements, the next important question to be addressed for Casa Vecchia was what concrete AAL functionality could we offer that would be useful for inhabitants of rural areas. From the broad range of functionality surveyed, monitoring activities of daily living (ADL), or functional monitoring [19], was most promising for our purposes. By the analysis of activity and the identification of typical patterns and significant deviations, monitoring can provide an enhanced level of safety and security for the elderly. However, this requires a balancing act between increasing safety, security and independence, on the one hand, and the perception of reduced privacy on the other. To achieve this balance, the activity observation in Casa Vecchia is based on environmental sensors only, which are smoothly integrated in the infrastructure of the participating households. Obtrusive devices, such as the cameras frequently used in AAL [1], are explicitly excluded, as are components that have to be worn on the body. These self-imposed restrictions limited our ability to track activities and the range of devices we could use, but they improved the impression, among our participants, of personal domestic privacy. The solution to the issue of privacy was, in the end, based on the methods presented by [5,12,20], whereas a specific focus was put on the measurement of inactivity [21]. That is, rather than monitor what seniors are doing, we monitored what they are not doing. In the next section, we discuss the algorithms that made that possible.

### 2.5. Data Processing and Distribution

There has been a steady increase in the number of news articles about elderly persons who have an accident and then wait helplessly for days until somebody recognizes that something is wrong. The observation of activity alone would not solve this problem and is, therefore, only one important part of AAL security features. What is needed is a further processing of the collected data, for example, passing them on to persons or organizations outside of the household who can intervene in case of an incident. The next obvious questions are: how much data should be passed on; in what format should it arrive; and to whom should it be sent? In our approach, we focused on giving clear and simple messages to each individual's informal caregivers. Although professional care giving and supporting institutions are important and although they participate in other aspects of the Casa Vecchia Project and many other projects, as well, there is a big segment of the elderly population who are still independent enough that they do not need permanent or professional support. With increasing age, this group experiences a gap between full independence, where no support is needed, and a certain degree of dependence, where professional care is required. For this group, it would not make sense to impose a contract with a professional care institution. The problem is that there is currently no business model that is sufficiently flexible and adaptive. The gap is currently filled by informal caregivers, who play an important role in supporting the elderly today [22,23]. Their importance will increase in the future. One goal in Casa Vecchia was to work within this existing framework of informal care. To that end, we endeavored to investigate the possibility of using ICT to support informal care givers. For example, consider the elderly person in the family context, in a circle of friends or in the neighborhood; groups that are currently under-represented in related research [22,23].

### 2.6. Usage and Interaction

Monitoring functions working quietly in the background may not be the only benefit for seniors living alone. It would be another enhancement of life quality to offer additional features with the ICT embedded in a smart home system, for example, for communication and information purposes. That said, current smart home systems often lack an acceptable level of usability [17,24]. Therefore, there is a specifically high demand for enhancements when aiming at supporting seniors in this regard. This is because the current cohort of elderly is known to be more reluctant to accept ICT than other age groups. On average, they have a lower level of computer literacy. Interfaces have to be usable without user training [17,25] and designed in a way that even computing novices are able to program them [19,26]. By lowering entry barriers, people would be motivated to explore the systems and, in this way, to increase their understanding and their interest in further enhancements [24]. This could have an additional benefit, because active contention can stimulate mental capabilities (as is the case in the application of serious games). Many smart home systems cannot fulfil such demands, because of high entry barriers. For example, they are based on classical concepts, such as the GUI, which requires a certain level of computing literacy. Therefore, they are not appropriate for AAL or, at least, not for the current generation of seniors.

Appropriate interfaces would also be of an economic benefit to AAL. Beside initial investments on the hardware installation procedures, adaptation and maintenance also contribute to the total costs of ownership [25] and have to be taken into account. Adequate interfaces enabling home owners or tenants to perform certain tasks themselves instead of requiring an expert for every small adaptation would help to reduce costs and increase comfort. However, as [6] point out, the probability is low that there would be a person who is willing and able to take over the role of a typical administrator in each household. Therefore, an appropriate combination of interactive and automated functions have to be provided. Advancements in the domain of entertainment electronics have shown possible alternatives to overcome such problems, in the form of automated functions. Today's TV sets can fully install themselves, search for channels and regional information, *etc*., requiring only a small amount of input from the user. This type of automation can, to a certain extent, also be considered for smart home and AAL systems. Components distributed in a home could configure themselves in a similar way with self-organizing network (SON) functionality. Further improvements in this regard can also be achieved by the application of artificial intelligence [27,28] to enhance control or programming tasks. The analysis of behavioral patterns, for example, could be used for programming devices. When it can be assumed that a device is not in use currently (because there is no activity recognized by a motion detector in the respective room), it can be switched off. Devices that are typically controlled coincidentally could be automatically combined into scenarios, so that explicit programming is reduced [29].

In Casa Vecchia, the issues of interactive and automated functions are addressed differently. Explicit interaction is enhanced by interfaces based on alternative UI concepts, such as informative art [30], Microsoft Bob or Magic Cap, which differentiate themselves from classical GUI by a straightforward, symbolic and metaphorical representation. Moreover, interaction is based on touch-based direct manipulation, which constitutes a practicable alternative to conventional GUI operation with a mouse and keyboard. Additionally, the system supports poly-modality, such as speech- and gesture-based interaction, which were prototypically implemented [31,32]. Artificial intelligence is used to automate processes. For example, pre-selection algorithms are applied to reduce the complexity and quantity of information provided to the user. News feeds are currently implemented as an exemplary function, which are filtered according to individual, implicitly communicated user preferences.

### 2.7. Related Empirical Research

Although there are a number of projects in AmI, smart homes and AAL (for an overview on the state-of-the-art, see, e.g., [1,33,34]) the problems addressed in this paper are not appropriately addressed elsewhere. Consider the unresolved challenges discussed in the previous sections related to technical, financial and interactional issues and a repeatedly voiced criticism of the mainstream research attempts in the past; the lack of real-world application. A considerable amount of research and development [17,18] has been performed in artificial environments, such as research institutions or laboratory settings (living labs), rather than in real-world contexts. The results therefore have a questionable ecological validity and may not be at all applicable to real-world settings [17,18,26]. The concepts and systems developed, “sound good in theory, but prove to be very difficult in practice”, as [26] concludes. However, the number of attempts at real-world AAL applications has increased in recent years. The majority are based on establishing new centralized care facilities or on enhancing existing care facilities with AAL technology. The benefits of centralization, such as an easy provision of an optimal infrastructure and a variety of adequate services, are unquestioned. Positive examples in this context are, for example, TigerPlace, Senior Care or Smarter Wohnen NRW. For an overview of projects, see, for example, [33,35,36].

What differentiates Casa Vecchia from those examples is that it is aiming at enabling seniors to prolong their stay in their current, familiar home. The reasons for this approach are that the vast majority of elderly people would prefer to stay in their current residence [11] and, as argued in the previous section, moving into nursing facilities would constitute a specific challenge for inhabitants of rural areas. Therefore, Casa Vecchia takes aging in place literally and enables seniors to stay in their current home. To be able to do this, we had to overcome the specific challenges in the rural area related to the adverse conditions, such as suboptimal Internet, power grid, public transport infrastructure, availability of qualified personnel (administrators, technicians), *etc*.

In summary, considering the potential importance of rural areas in the context of AAL, it was surprising that only a small number of researchers have addressed the specific combination of AAL, smart homes and rural areas that have been in our focus in the Casa Vecchia Project. The work of [37], for example, addresses technological aspects related to AAL in rural areas with a focus on connectivity (e.g., the availability of broadband Internet). Another example is [38], who present a PDA supporting AAL in rural areas with an emphasis on tele-healthcare. The focus on healthcare is also central in the project, EMOTIONAAL (emotionaal.eu), as well as in a small number of other activities referred to by [33]. The project, Seniorlab (www.seniorlab.es), constitutes a living lab that aims at fostering the development of AAL solutions for inhabitants of rural areas.

Casa Vecchia differentiates itself from those examples, because it is a multi-purpose platform that already includes a set of exemplary functions (health support, communication, comfort), and it can be enhanced with a broad variety of functionality and is adaptive to the changing needs and requirements of the users.

### 2.8. Casa Vecchia Requirements

The thorough investigation of related theoretical and empirical work, as well as their outcomes and solutions discussed in the previous section built the foundation for Casa Vecchia. The following set of functional and non-functional requirements were derived as guidelines for the project:

#### 2.8.1. Non-Functional Requirements

- The basic system of Casa Vecchia supporting AAL in a rural area should be integrable and adaptable to the arbitrary living environments of elderly people;
- Interference with the existing infrastructure and familiar processes should be as minimal as possible;
- The system has to be affordable in terms of both initial investments and running costs;
- The target group is independently-living seniors, who do not yet need professional or continuous support;
- Participants can draw on a functioning social network, including informal support from family, friends and community members;
- The system is characterized by a high level of usability, adapted to the needs of the target group.

#### 2.8.2. Functional Requirements

- The main functionality is to increase the security and safety of the users;
- Security functions are automated and do not require active user interaction;
- Additional interactive functions should support the enhancement of the comfort and life quality of the users, (e.g., communication, e-inclusion);
- Control, maintenance and configuration by the users themselves should be supported;
- Continuous enhancement of the system and adaptation to changing needs is ensured.

## 3. Methods

On the basis of the relevant related work and the derived requirements, the Casa Vecchia Project was conceptualized and carried out. The outcome was thoroughly analyzed afterwards, as will be described in detail in Section 4. Casa Vecchia is defined as a feasibility study performed under real-world conditions and, therefore, differentiates itself from the experimental and controlled studies enumerated in the previous section. The theoretical aspects discussed in Section 2 build the basis of such an approach. A stable technological basis is a prerequisite to be able to run AAL functions, such as data collection and analysis. This is necessary to differentiate between normal and unusual behavior. An appropriate communication infrastructure is needed to transfer data to the outside, in order to signal for help. Explicit interaction with ICT can have additional benefits for the elderly, in terms of inclusion, independence and well being. However, this requires appropriate and usable interfaces. In contrast to the controlled conditions of labs or model houses, the establishment of such basic functionality constitutes a big challenge and requires a certain degree of customization. These characteristics of the study itself and the study sample are therefore also different from typical experimental studies performed under controlled conditions. This also required a different approach to a purely hypothesis-driven one based on standard evaluation and analysis methods and the achievement of generalizable results. Methodological guidelines and quality criteria were taken into consideration; the study followed a phenomenological approach with a focus on qualitative methods [39,40].

### 3.1. Characteristics of the Study Sample

Based on the available financial and human resources for the project, the number of possible field installations was about 20 households. The target group for the project were people above the age of 60, which has a size of around 50,000 persons in Carinthia. Because of the expected reluctance of the elderly towards ICT, the contents for advertising the project with the target group were intensively reviewed by researchers of different disciplines, as well as representatives of the target group. The announcements were made via conventional media channels, such as newspapers and local radio stations known to be popular with the respective age groups, as well as institutions frequently visited by the target group, such as social insurance companies and care institutions. Other channels, like word of mouth, announcements at the university and the Internet (email, social media, web), were also used. Fifty people volunteered, a number representing roughly one one-thousandth of the target population. In the end, only about half of them proved both willing and suitable to participate. We had to exclude, for example, people who were already suffering from a chronic disease that required professional support. Because of ethical reasons, their participation was politely refused. It was also made clear to the other potential participants that the system used in the project is a prototype system and not an appropriate replacement for a professional care system. We were also clear in stating that the system could not be guaranteed to react correctly in case of an emergency. Another reason for exclusion was when it turned out that people just were interested in the provision and installation of smart home equipment, but not in the problems addressed in the project. When they refused to allow data collection and to participate in interviews or to fill out questionnaires, their participation was ended. The complete information regarding the goals, limitations and disclaimers were presented to potential participants in the form of a written informed consent, which they had to sign to be able to participate in the project.

**Figure 1.**
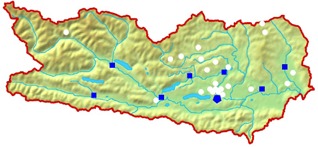
Topographic distribution of participating households. The white circles represent the participating households. The blue rectangles represent the bigger cities in Carinthia (ranging between 10,000 and 60,000 inhabitants); the polygon shows the location of the university (where the server infrastructure is hosted) in the capitol of Carinthia (which has around 100,000 inhabitants.

Finally, 22 households, scattered across several rural regions of Carinthia, could be included in the project. Two of them dropped out in the course of the project. The topographic distribution of the involved residences is shown in Figure 1. The majority of buildings are family houses; only two participants lived in apartments. The age of the buildings ranges from a three hundred year-old farm house to a low-energy house built only a few years ago. Most are family homes, built between 1960 and 1980.

The residences are inhabited by either one elderly person (in this group, the majority is female) or a heterosexual couple. The age of the participants ranged from 50 to 83 years. The youngest participant was a woman who was already retired because of severe health problems in the past and informed about the project via word of mouth from another participant. Although she did not fit in the defined age range, all other requirements were fulfilled, and she was highly motivated to participate. Together with each participant (or participating couple) a trusted person was involved in the project. This person is an informal caregiver, such as a relative, a good friend or a neighbor who has already been supporting the participating senior before the project was started. An overview on the demographic characteristics of the participants is given in Table 1.

### 3.2. Hardware Implementation

**Figure 2.**
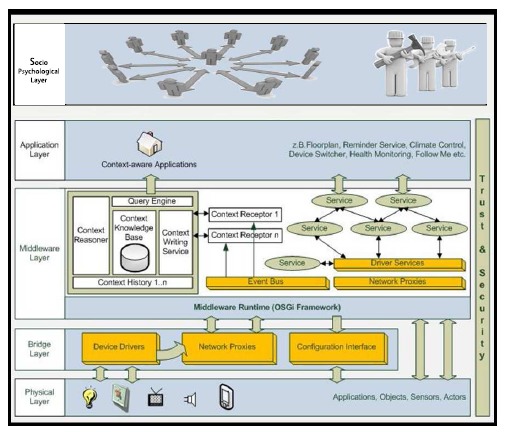
Layered OSGi architecture, originally developed by [41] to support the research group's activities on a smart home, in general, and AAL, in particular.

The technical platform of Casa Vecchia, shown in Figure 2, is based on OSGi and was initially implemented by [41] and continually further developed within the project. It is designed as a layered architecture in which the bottom layer manages hardware components, such as actuators and sensors, which control or receive status information from the devices to which they are attached. To overcome the problem of incompatibility and interoperability discussed in Section 2, a bridge layer manages abstracted, hardware-independent representations of the component/device combinations connected to the system. With this abstraction, it is possible to integrate components from different manufacturers, based on different connection types (wired, wireless) or other deviating parameters (e.g., binary or discrete switching states). With this architecture, it was possible to integrate components from diverse smart home systems, such as digitalSTROM (www.digitalstrom.org), a power-line system originating in Switzerland, Homematic and FS 20 (www.homematic.com), German wireless systems, and custom components built on the basis of Arduino (www.arduino.cc). In the middleware layer, the abstracted device representations serve either as service providers (e.g., sensors observing the status of an attached device) or service users (e.g., an actuator using twilight sensor information to decide whether outside lights should be switched on). The presentation layer enables user control and interaction with the system via customized interfaces, which are adapted either to the type of device (e.g., desktop PC, tablet or smartphone) or the type of task (configuration, administration or basic control). As an addition to the initial architecture of [41], a virtual layer is introduced to represent components that go beyond the technological foundation, such as social network (e.g., informal caregivers, such as relatives or friends), institutions and public authorities (potentially relevant for financing and funding) or enterprises (e.g., local, skilled craftsmen, important for installation, system integration and maintenance).

**Figure 3.**
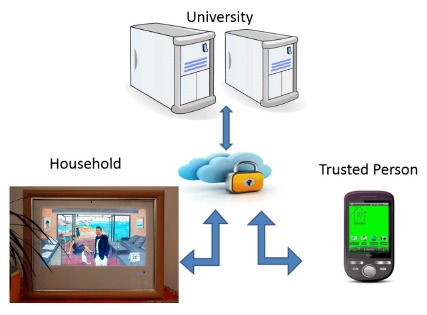
The system architecture showing the servers at the university responsible for backup, data analysis and transfer (**top**), the central unit in the participating household (**bottom left**) and the smart phone of the trusted person (**bottom right**).

The on-site installation of the system in the participating households was performed following a phase model similar to the approach described in [24]. In parallel, connections to servers at the university (see Figure 3) and to the caregiver were established. This enabled the appropriate and distributed observation of daily activities, deviations and potential incidents in the residences of the participants.

Within the first phase (requirement elicitation), the topographic and technical features of the households and relevant information about the participating persons (the seniors and their informal caregivers or trusted persons) were surveyed, and the procedure for the roll-out was defined. In the second phase, initial installations adapted to the surveyed data took place, and basic features were configured with a focus on the security functions.

Because of the necessity to retrofit the system into real living environments, several constraints had to be faced. As the majority of households were old buildings with limited possibilities for additional wiring and taking into account the cost aspect, only a wireless or power-line system was used. Because the power-line system, digitalSTROM, did not provide a reasonable variety of functionality at the time the project was started, the wireless system, Homematic, was chosen. The system provides an adequate functional range and flexibility, as well as an interface (XML-RPC), which allowed integration into the platform and adaptation to the different circumstances. Moreover, the price level for the required features is around € 1000 per household, which is acceptable. About the same amount of money had to be calculated for the installation, which was done in cooperation with certified electricians. Because of potential malfunctions and the need for maintenance, small and medium enterprises (SMEs) whose workshops are near the participating households were selected. In the last phase, the system in the households was continuously fine-tuned and adapted according to either the needs observed by Casa Vecchia team members or to those explicitly expressed by the participants themselves.

The final status of installation is shown in Table 2. The number of installed devices altogether is around 150; the mean number of devices per household is 7.

### 3.3. Data Acquisition and Processing

The goal of Casa Vecchia to support the prolongation of independent living had to be put into practice very cautiously. The advantages of being able to stay in a familiar home for a longer period of time should not be reversed by technology that overexerts people. To keep the system as unobtrusive as possible, we tried to install components only into areas where they would not invade privacy, but would still be appropriate for the monitoring of daily activities. The participants helped identify areas fulfilling those requirements, and the components were installed accordingly. The goal of unobtrusiveness was also taken into account by installing components attached to devices in such a way so as not to interfere with habits and daily routines. This was achieved by adapting the smart components to the existing devices, even if the latter were already outdated from a technical point of view.

The goal of a systematic integration and positioning of devices was to be able to observe activity at a reasonable quality. The rule of thumb was to receive an activity-related event at least once per hour. This frequency should enable the differentiation of normal activity from significant deviations and, in this way, made it possible to react to potential problems within a reasonable time frame. This was a challenge, because activities that take place in the household can differ significantly in sequence and routine. Additionally, differences in floor plans of the participating households, the different constellations of devices and their usage had to be considered in the conceptualization of monitoring daily activities. Within the initial phase of the project, the participants were therefore asked to describe their typical day; what activities are performed and what devices are involved in those activities. For example, if a participant reported preparing coffee every morning, a sensor (e.g., DS (device sensor); see Table 2) was attached to the coffee maker. Whenever the device is used, an event is triggered and collected by the Casa Vecchia platform. If the typical activity of a person would be to move from the bedroom to the bathroom, a motion sensor (MS) was installed on the corridor between these two rooms, also triggering an event. In that way, components of the system were either positioned in central locations (e.g., motion sensors), integrated in the wiring or attached to specific devices according to the descriptions that the participants gave. In this manner, we built each system to reach the intended average frequency of one event per hour. As shown in Table 3, the combination of devices and their allocation to daily activities is quite heterogeneous. However, the different combinations of components are used to achieve the same set of goals; to receive events at a reasonable frequency, but in an unobtrusive way. An overview of the devices installed and to what time of the day they typically correspond is shown in Table 3.

In parallel to the on-site installation in the households, a server infrastructure managing the data exchange between elderly participants and trusted persons was established at the university (shown in Figure 3). The server manages and transfers activity data from the connected households over secure channels and performs backups of the data at a central and secure location. For privacy and security reasons, no detailed information of the households (location, floor plan, segmentation, *etc*.) or personal information of the participants are transmitted. Only device identifiers, time stamps and status information are received and locally interpreted on the server. For example the message “HEQ00012345, 07-12-12 07:30:01, On” means that a sensor was activated at half past seven in the morning. In combination with metadata stored on a separate logical server, it can be determined that this sensor is attached to the coffee maker in a certain household. The arriving data is further analyzed by interpreting the frequency, time of occurrence and the typicality to derive meaningful status information that is transmitted to the smart phone of the trusted person.

**Figure 4.**
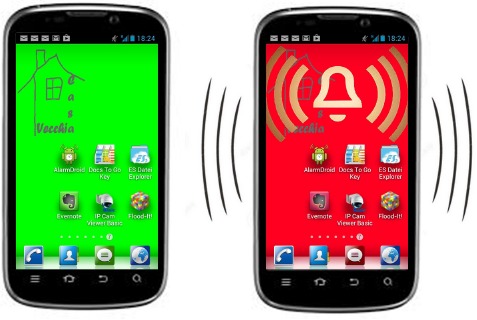
Smartphone of trusted person with different background colors and alerting signals.

To guarantee an appropriate level of privacy, the trusted person also does not receive detailed information about which concrete activity is taking place in the household or when the activities occur. The only information provided is the overall status, illustrated via changing background colors on a smartphone, as shown in Figure 4, using a traffic light metaphor.

A green background represents a normal situation. When the background changes to yellow the activity level in the connected household has fallen below a certain threshold value. When the color changes to red, either the threshold indicating normal behavior has not been met or a device signaling a dangerous situation (such as a smoke detector) has been triggered. In the latter case, additional cues (an alert sign and vibrations) would attract the attention of the user. The status information is implemented as an Android background widget (other platforms are in progress), similar to background widgets that provide weather information. This way, the changing color of the background does not interfere with the usage of the other functions of the smartphone.

### 3.4. Security Features Based on Activity Recognition

The actual release of the smartphone widget automatically recognizes activity deviations based on statistical analysis. The basic algorithms work as follows: In the first step, each day of a week is segmented into time slots *x_i,j,k_* of equal length, whereas index *i* represents the current week (0, …, *number* of investigated weeks from historical data), index *j* the day of a week (0, …, 6) and index *k* determines the actual timeslot (0, …, 23 if slots of 1 h are applied). For each timeslot *x_i,j,k_* , the occurrence of events starting from the current week *i* back to week *i* − *m* is analyzed, where a timeslot including at least one event is marked as active (1) or inactive (0) if no events where recorded:

$$f(x_{i,j,k})=\{\begin{array}{ll} 1, & \text{if}\;x_{i,j,k}\;\text{contains events}\\ 0, & \text{else} \end{array}$$

Thus, on the basis of historical event data from *m* weeks, the probability *p*(*a_j,k_*) of activity in a certain timeslot *k* on weekday *j* can be calculated:

$$p(a_{j,k})=\frac{1}{m}\sum_{k=1}^{m}f(x_{i,j,k})$$

A calculation for the weekday, Monday, segmented in timeslots of 1 h is shown in Table 4.

On the basis of these data, a threshold value can be set individually for each household to trigger the alarming function of the smartphone application (typically *p* ≥ 0.9). The probability of activity (and triggered events) is generally lower at night time and, therefore, below the threshold. Therefore, the background of the trusted person's smartphone would remain green even if no events were recorded. Conversely, if no events have been recorded in timeslots with a high probability of events, then an alarm is triggered.

### 3.5. Interaction

**Figure 5.**
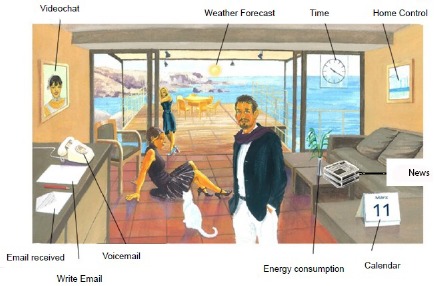
Overview of available interactive features.

As mentioned, unobtrusiveness was a central goal of the installation. There was only one exception, the intentionally intrusive central unit (a redesigned embedded PC) of the Casa Vecchia system, shown in Figure 5. We asked to be allowed to mount the unit at a location that is easy to access and where the participants would frequently pass by during the day, because the device should attract attention and invoke the curiosity of the participants. Motivating them to familiarize themselves with ICT could enhance the quality of life of the elderly, by enhancing communication and supporting e-inclusion, for example. The range of functionality can go from simple standard use, such as web surfing and email, to complex functions, such as health services, programming, configuration and maintenance, as discussed in Section 2. However, a certain level of reluctance was expected, and therefore, one of the research questions was: what influences the acceptance of of ICT in the home? To overcome entry barriers, the system was designed with the requirement of a high level of usability. The resulting design combines features from the concept of informative art [30] to give the interface an appealing, artistic, instead of a possibly repelling, technical touch. Furthermore, the unit was integrated in a picture frame, making it look like a digital picture rather than a conventional PC. Interface metaphor concepts, such as Microsoft Bob and Magic Cap (e.g., writing desk, room, bookshelf), served as examples for the design of functional elements. The interface, implemented by [42], is shown in Figure 5.

**Figure 6.**
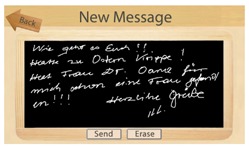
Handwritten (e-mail) message.

The functions are designed as metaphorical symbols, for example, a white sheet of paper together with a pencil lying on a sideboard symbolizes written messages. An example message written by one of the Casa Vecchia participants in the course of the project is shown in Figure 6.

Usability was ensured in such a manner that the steps not directly relevant to a task are hidden from the user. For example, parts of the operating system, such as boot up messages or login, do not trouble the user. To write an e-mail, for example, the users simply have to point a finger at the respective part of the screen. A virtual chalkboard appears on which a message can directly be written, either with the finger or with a stylus. After having pressed the send button, the message is directly distributed as a graphics attachment to the email address of the addressee. In this way, the participants could write e-mails without having to struggle with elements and procedures that they may not be familiar with, such as having to select a subject, addressee, priority level, *etc*. The written message is further processed by an e-mail application automatically distributing it to the trusted person or other e-mail addresses pre-registered with the system. The participants also could receive email messages. This feature was embodied in the envelope lying on the virtual side board next to the paper and pencil. By clicking the envelope, the received email was displayed; with the possibility to turn pages if more than one email was received.

Other available functions are news feeds and weather forecasts displaying the data of freely available web weather services. News is extracted from RSS feeds provided by different media content providers. A basic set of RSS feeds is provided containing a pre-selection of news that fit the interests expressed by the participants during the initial interviews. The news includes, for example, regional information, information on national politics, science, sports or celebrities. To enhance the usability of this service, recommender technologies have been integrated. The server located at the university (see Figure 3) is connected to the news recommender system developed at the Graz University of Technology [43], which is analyzing the interaction of the participants with the news feed in real time. Every time the participants access the news feature, the recommender system analyzes the usage behavior and calculates a value of interest for the respective news categories. The more often a certain category is accessed, the higher the value. The differences in interest affect the presentation. More popular categories are characterized by a bigger icon size, similar to the design of tag clouds, where keywords of higher importance are bigger than those that are less important. Other functions were continuously added or enhanced, based on, for example, the demands of the participants. An example is a door camera feature. Participants were alerted by commercials and news about the possibility of ICT-based observation of a house and asked us about the respective possibilities. This feature was integrated in some of the participating households with a webcam installed on the front door, with a video feed that could be observed on the central unit of the system. This enhanced comfort, because the participants did not have to go personally to the front door, but could observe the area remotely. Other features, such as motion detection, recording features and observation with a smart phone, were continuously added, as well.

### 3.6. Socio-Psychological Methods

The different phases of hardware and software installation were accompanied by a sequence of socio-psychological inquiries carried out in three waves on the basis of standardized questionnaires containing the categories enumerated in the list below. The inquiries were based on data collection on different aspects that are described in the related literature as being relevant in the context of AAL. Those aspects were summarized into the following categories.

- Demographic aspects: personal biography, demographic background;
- Lifestyle: attitude on life, interests, life satisfaction;
- Technology: access to technology, technological experience, technology acceptance;
- Project: interest and motivation for project participation, expectations.

On an implicit level, socio-psychological evaluations involved behavioral observations. For example, it was observed whether the system was used or not, which components and features were in use and at what frequency. The focus of this paper is on technological aspects. A detailed discussion of socio-psychological methods and goals are given in [44].

## 4. Results

The central focus of the Casa Vecchia project was the investigation of the feasibility of AAL in rural areas. Results could be achieved on a qualitative, as well as on a quantitative level. In the following section, the results are discussed in the following categories: technology related aspects; aspects related to data processing and activity tracking; and aspects related to interaction and socio-psychology.

### 4.1. Technology-Related Results

In experimental settings, such as living labs, an adequate basic infrastructure can be taken for granted; for example, the electrical wiring conforming to state-of-the-art standards. The same applies for the availability, performance and robustness of Internet connections. Therefore, these aspects are often left out of the discussion of related literature. In the real world, rural settings of the Casa Vecchia project, we had to deal with these fundamental aspects. For example, electrical grounding was not adequate or did not even exist, and wiring did not correspond to the floor plan. Moreover, wires did not comply to international coloring or size conventions. This made it more difficult and more expensive than expected to retrofit the components and sometimes even dangerous. Suboptimal Internet connectivity, resulting in unstable and slow connections, was another problem. Strong efforts into self-healing and automatic boot-up had to be invested to prevent the team members from having to drive for hours just to reboot a system. A reasonably robust system could finally be achieved by enhancing and fine-tuning the features of the Ubuntu Linux operating system.

**Figure 7.**
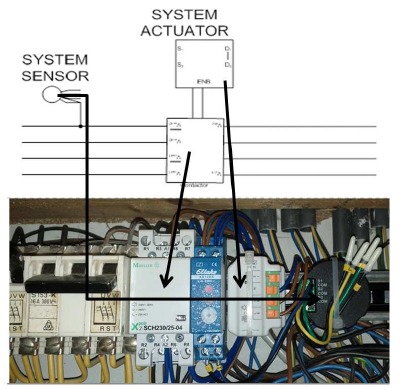
Kitchen stove coupled sensor/actuator device.

Other circumstances requiring customization were, for example, related to the fact that smart home systems in the low-price segment are not, by default, capable of dealing with specific technical requirements, such as voltage. Kitchen stoves in the region typically operate with a 380 V, three-phase current instead of the standard 230 V, one-phase current. Because of the fact that the stove is one of the most potentially dangerous devices in a household, it must be integrated into the security features of an AAL system. However, to achieve the goal of unobtrusiveness, this integration should not interfere with the mode of operation the participants are familiar with. This differentiates our approach from solutions available on the market. These other solutions sometimes require changing familiar procedures, for example, pressing additional buttons before being able to use the stove (see, for example, www.cookstop.com/products.html). Not surprisingly, meeting all of these requirements is quite complex. The Casa Vecchia solution (shown in Figure 7) is a combination of four components installed in the household's central fuse box. One component is tracking the electric flow and informs the second component about whether the stove is on or off. The third component enables switching a 380 V current to 230 V, which even a cheap smart home system can deal with. The fourth component can automatically cut off the current supply to the stove, in case of emergency. If, for example, the stove is on and a smoke detector in the vicinity is triggered, the stove is switched off automatically to avoid serious damage. The improved level of security is equal to systems available on the market, but the usability and comfort is greatly improved. Consider that the familiar usage patterns do not have to be changed and, in the best case, the participants do not even notice that additional components are attached to their devices.

By customizing the installations in the way described above, it was possible to integrate even exotic or outdated home appliances. This is another problem that smart home systems available on the market cannot deal with. For example, in one household, we could establish a light corridor in a cellar with a very complex floor plan or integrate a water sensor into our system, similar to an example presented by [18]. Additionally, custom devices, such as a floor pressure sensor informing about whether someone is standing in front of the entrance door or using the accelerometer of a smartphone to dim lights, could be realized. However, they did not reach a satisfactory level of reliability to be deployed and are therefore currently undergoing further development.

Another hardware-related finding had not been anticipated, in terms of either occurrence or relevance. Many smart home systems use buttons instead of conventional switches. In principle, buttons provide the same functionality as switches, with the difference that their rocker does not stay in the position to which they are switched. When pressed, they send an impulse, and then the rocker moves back to its original position. This small technical difference had a big effect on the interaction level, because it made it impossible for the participants to observe the current status of the button itself, as well as the device(s) it controlled. The feedback they got from the rocker position of conventional switches by which they could observe the status of a controlled device (for example, an outside or cellar light not directly visible) was no longer available. In Casa Vecchia one of these buttons played a central role. It was used to switch off all dangerous devices (e.g., stove, iron) together, for example, when leaving home for a longer period of time. The big advantage of this kind of button is that they can be mounted at any position, because they are wireless and battery operated. In our case, the button was put in a prominent position next to the entrance door. However, the missing feedback led to the problem that participants either did not remember to press it when they left or forgot to switch it on again many times when they came back home. Sometimes, when the participants tried to cook, the stove did not react when turned on. In the starting phase of the project, this resulted in frequent calls to the local electricians, as well as to the Casa Vecchia team. Later on in the project, the participants became aware that they probably had forgotten to press the button when they came home and the stove was just disconnected from power. Meanwhile, some participants put sticky notes on the entrance door or the stove to remind themselves to check the button, but this completely contradicts the understanding of unobtrusive technology.

Although the all-off button has this drawback, on at least one occasion, it may have saved a participant from harm. This participant started to cook lunch, but then, he saw that an important ingredient was missing. He spontaneously left the house to get the ingredient from the grocery store in the next village around five kilometers away. Before leaving the home, he remembered to press the all off button. When coming back and entering the kitchen, he became aware that he had left a pan with oil on the stove and that the stove had been left on. Within the 20 min it took him to visit the grocery shop, the kitchen would probably have caught fire had our system not been present. Even if he had forgotten to press the button, the coupled stove device shown in Figure 7 would have switched off the stove in time, triggered by the attached smoke detector.

### 4.2. Results Related to Activity Tracking

The goal of being able to track events at a reasonable frequency (one event/h) could not only be reached, but exceeded. The data collection resulted in a final number (as of February 2014) of about 2.5 million events, with an average of around 100,000 events per household, which means that, on average, four events per household per hour were recorded. Detailed information is shown in Table 5.

**Figure 8.**
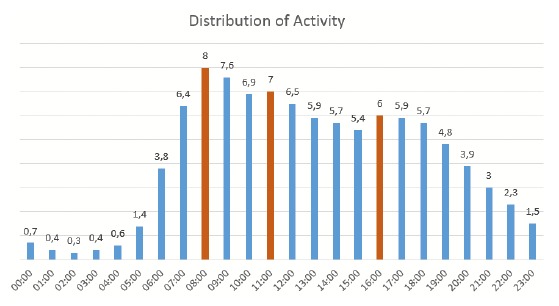
Distribution of events over a day in all households (relative percent) with clearly observable activity peaks in the morning, around noon and in the early evening.

In the next step, the collected data was investigated further. For example, daily activity patterns and deviations were analyzed in detail. Figure 8 shows the overall distribution of recorded events from all households over the period of 24 h.

**Figure 9.**
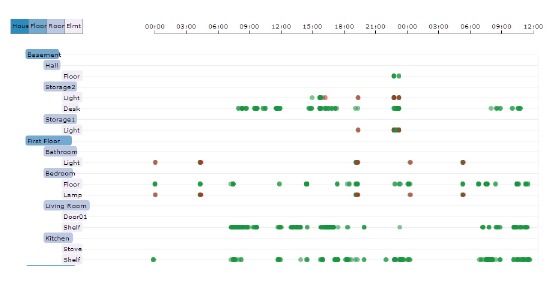
A snapshot from a period of 36 h in one household. The devices, their position in the household and their activation patterns are shown. Devices that have two person-triggered states (e.g., can be switched on and off) are represented by dots with two different colors (green = on; and red = off). Devices that are only triggered in the on state, for example motion sensors, only have a green color (on), because they are switched off automatically.

Peaks are observable in the morning, around noon and in the evening. In contrast, night-time shows periods of low activity. These results were, for example, taken into account to fine-tune the thresholds of the smartphone application described in Section 3. To get an impression of what kinds of events are occurring and in what sequence and frequency the devices are triggering, Figure 9 shows a timeslot of 36 h from one household containing 1068 events. Information about the type and position of the components is given and, if the device is capable, of different states. For example, a light can be manually switched on and off (therefore, the status is visualized by green = on and red = off). A motion sensor only detects movement triggered by a person (green = on), but is switched off automatically after a predefined delay time. Therefore, only the on state is represented in the graph, *etc*.

**Figure 10.**
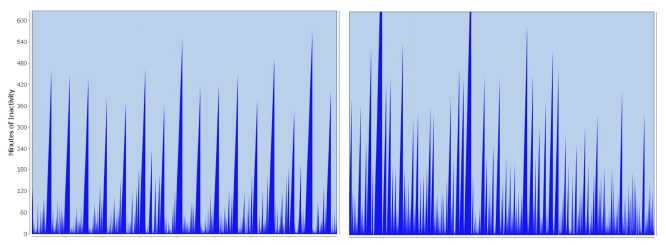
Inactivity patterns of two households.

The biggest difficulty has been to develop generalizable algorithms, because of significant differences in the course of the day of the different households. The charts shown in Figure 10 illustrate the problem on the activity patterns from two participants (both single, living alone, in family houses).

The peaks in the charts represent inactivity, because this dimension is important for estimating an incident or triggering an alarm. The higher the peaks, the longer the inactivity. The information is derived from the events triggered by the components installed in the corresponding household. When a device has triggered an event (e.g., the coffee maker is switched on) a peak is initialized and proportionally grows on the *x*-as well as on the *y*-axis until the next event is perceived. Then, the inactivity level falls down to zero (no inactivity = activity), and a new peak is initialized. If a peak exceeds a certain height, which means that the inactivity is longer than usual (as described in Section 3.3), an alarm is triggered. Looking at the two example charts, it can be observed that the left chart is characterized by a high regularity. The highest peaks represent night-time, when the person is regularly inactive for around 8 h, and clearly distinguish themselves from the other parts of the chart. During the day, the frequency of traceable events is in a range between 60 and 120 min. These regularities make it easy to automatically identify significant deviations and define critical thresholds in the corresponding pattern.

In the pattern shown on the right of Figure 10, the situation is different. No obvious regularities can be observed; periods of activity and inactivity seem to alternate randomly, making it more difficult to correctly identify significant deviations. However, the deviation recognition described in Section 3 reached a satisfactory quality level, and advanced algorithms developed by [45] based on the work of [21] could, for example, demonstrate how the system would differentiate between standard and deviating patterns. The algorithms were under continuous development, taking into account not only activities occurring on a daily level, but also differentiating between longer time periods, such as weeks or months, in order to reflect routines (e.g., weekly meetings in a club or getting up later on weekends). Currently, the computations include data from the previous three months, allowing for adaptation to changes in daily routines that depend on seasonal deviations. For example, people typically spend more time outside in summer. Specific analysis of the data can also be performed, but this work is currently in progress. Another example is the usage of the kitchen stove, which contributes to activity tracking in general. Data from the stove can also be used for the evaluation of specific areas, such as nutrition. The same applies for data collected from devices, such as the water sensor, which was integrated into the smart system in one household. The recorded data can be used for general activity tracking, as well as for the analysis of hygiene.

A problem related to activity tracking, or more concretely, to the data transfer to the trusted persons, occurred in the starting phases. An initial version of the smartphone widget was launched for the purpose of testing the principal functions of the platform, as well as the connectivity and stability of the data transfer. Because of several unknowns and missing historical data for threshold estimation, the displayed status information was not based on real-time probability calculation, but on manually-set thresholds. According to information the participants provided in the initial interviews regarding their typical activities and routines, a rule of thumb was applied to estimate the time for changing the background colors from green to yellow and red. For example, if the typical time between two activities was 1 h, the threshold for changing the background color from green to yellow was manually set to 60 min. When activity patterns changed, seasonally, for example, the thresholds would have had to be re-adjusted again manually. This rigid configuration (as well as technical problems, such as power outages and system shutdowns) made the smart phones change the background into red quite often. At first, this irritated the trusted persons, and they called the participating seniors or members of the Casa Vecchia team. After a certain period, the trusted persons got used to this problem, but this resulted in a negative effect. Given the low reliability they had experienced, they learned to ignore the changing background colors of their smart phones, because it was impossible for them to differentiate whether there is a real problem or just a technical one. In parallel to the continuous automation of the threshold configuration described in Section 3, the color scheme of the smartphone was therefore also enhanced with additional states. If the connection between server, household and smartphone was disturbed, the display did not show a background color, but displayed question marks instead. Moreover, when the seniors left their homes and informed the system by pressing the all off button, the background color of the smartphone changed to light blue. With the introduction of these new states and enhanced precision of the basic algorithm, the trusted persons re-gained confidence in the system. In the final phase, the precision of activity tracking could be increased again to a degree that even surprised the participants. The seniors sometimes received a phone call from their trusted persons because of a significant deviation in their typical behavior, such as chatting for an unusually long time with a neighbor in the garden.

### 4.3. Results Related to Interaction

The smart home components were evaluated regarding the potential to involve users in programming, configuration and maintenance tasks. The components of the system are not automatically configured, but require a sequence of manual steps for coupling and configuration comparable to the procedure of pairing Bluetooth devices. The first step is to bring the components into pairing mode. In most cases, this is pairing them with a gateway module connected to the central unit of the smart home system. The problem with the pairing mechanism was that the steps differed incomprehensibly from component to component. On some components, it was necessary to activate pairing mode by pressing a button, then an LED signaling pairing readiness flashed. On other devices, it was necessary to hold the pairing button for several seconds (up to ten), and then an LED gave feedback. The colors of the LEDs differed: some of them were only able to flash in one color; others were flashing in different colors (red, green, yellow) signaling different states. Some devices did not even have a feedback LED. The installations and configurations were completely done by the Casa Vecchia team members in order to avoid confronting laypersons with such complex heterogeneity.

The goal of involving end users by stimulating interaction with the system's central unit could be achieved to a certain extent. The participants liked the design and the simplicity of the features. Most of them frequently used the features, such as the weather forecast. The news feed feature was specifically of interest for participants living in very remote areas where the daily newspaper is only delivered in the afternoon. They then are already informed about current affairs from radio or television and, therefore, are no longer interested in reading the newspaper. The news feeds provided a good alternative for reading the news early in the morning.

One indicator that this approach worked out very well was that some of the participants even made the system a part of their daily life and were disappointed when it did not work properly. Then, they called the Casa Vecchia team and informed us that the calendar displays a wrong date or that the weather feature does not correctly show the estimations of the coming days. They tried out all of the available features intensively; many of them made proposals for additional ones (such as the door camera), and many of them also demonstrated the system to visitors. Specifically, visiting children seemed to love the appealing design and tried out everything simultaneously, sometimes resulting in the system freezing. However, this was also a good reality check for our system. Most of the time, the participants simply had to pull the power plug and plug it in again after a few minutes. In particular cases, the motivation to use the system worked even better than expected. People who were not writing emails before participating in the project began writing short, hand-written messages. At first, this was infrequent, but, because their contact persons (children and grand children) welcomed the new possibilities of interacting with them, they were motivated to use this feature more intensively. This made it necessary to enhance the feature, enabling them to choose between different addressees and to increase the amount of text to be written. For this requirement, the handwriting feature was not adequate; therefore, it was enhanced with the possibility to use a keyboard and a mouse in order to write full-sized emails. Table 6 shows an overview of the usage frequencies of the available features, which did not change significantly over time.

**Figure 11.**
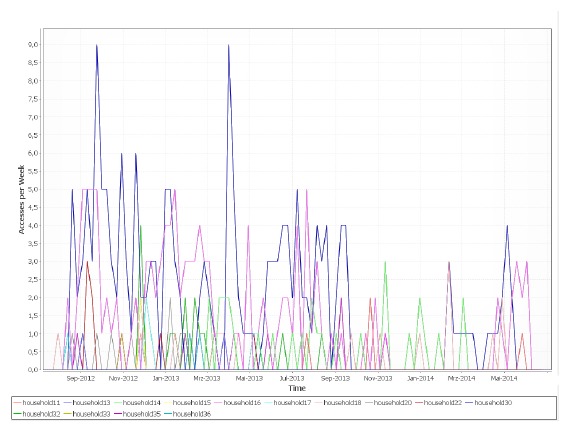
Frequency and temporal changes in the usage of the weather feature.

Figure 11 shows the development of usage activity on the weather feature over a period of 18 months. The success of the stepwise addition of functionality supports the findings of [24] that, for lay persons, it may not be clear in the first place what technology can do for them. The provision of exemplary functions motivated users to think of additional features and increased their interest in further enhancements.

In parallel to the field installations, a lab study was carried out with the goal of investigating alternative interaction methods, based on natural speech and gestures. On the basis of the Casa Vecchia platform, functions were implemented that would enable users to control their smart home with speech or gesture commands. The results are further evidence of the quality of the Casa Vecchia platform. In a controlled study, a sample of 32 participants were asked to perform nine tasks controlling smart home appliances, without familiarization (after a 10-min introduction). The tasks were to switch on and off lights or a radio and to control blinds (open, close, open more, open less), and the participants had three attempts to perform them. As said, without being familiar with the system, 55.9% of the participants could correctly perform the voice-based tasks on the first attempt, and 87.1% of them succeeded by the third attempt. In the gesture condition, 64.8% were successful in the first attempt and 91.6% by the third. For a detailed discussion, see [32].

### 4.4. Results Related to Socio-Psychological Aspects

About one hundred formal and informal interviews and contextual inquiries were carried out in different constellations (with the elderly person alone, together with the trusted persons, with the trusted persons alone, *etc*.) and a large amount of data was collected. The focus of this paper is the technological feasibility; therefore, the emphasis of the following sections is put on the technology-related results, and only an overview of the other findings is given.

The socio-psychological approach was a phenomenological rather than a hypothesis-driven one. We tried to observe the life of real people in their real living environments and their social network and to induce a theoretical framework from that data according to the approach of grounded theory [46].

The categorization enumerated in Section 3.6 therefore only served as guidance, but was not intended to limit the thoughts and opinions of the interviewees. Explorative and statistical analysis were performed yielding significant results and revealing clear tendencies, specifically regarding the access to technology and technology acceptance. Participants already had a low level of experience with computers. At least, they had used systems, such as the AS 400, in their past work. The range of computing knowledge is broad and goes from playing solitaire on an outdated PC, through using a state-of-the-art laptop for writing emails and surfing the Internet frequently, to possessing and using a smart phone. Because of this experience, the access to technology and technology acceptance in the sample is, not surprisingly, positive. That said, across our sample, gender had a strong correlation to attitude regarding technology. Whereas the men in the sample have a generally positive attitude toward technology, the women clearly differentiate between technology satisfying concrete needs or solving acute problems and other technology that is just for “its own sake” and, therefore, not of interest to them.

**Figure 12.**
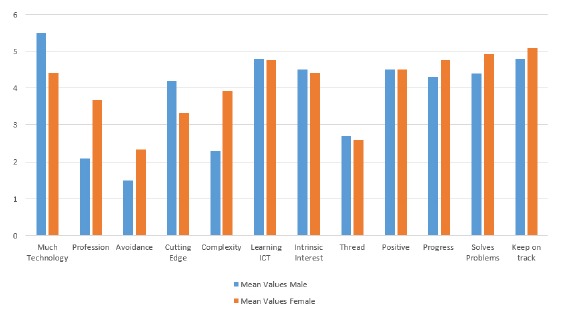
Mean values of the technology-oriented questions, ranging from 6 (strongly agree) to 1 (strongly disagree).

These differences are clearly observable in the responses to the technology related of the provided questionnaires. Figure 12 shows an overview of the results (N = 21, male = 10, female = 11). The phrases rated by the participants are:

‐ I always had to deal with technology in my life;
‐ A profession in the technology sector would not have been adequate for me;
‐ I was avoiding technology whenever possible;
‐ I was always interested in possessing the newest technology;
‐ Complex technology always irritated me;
‐ I liked to learn ICT skills;
‐ I was interested in understanding new and advanced devices;
‐ Technology is more a threat than a benefit;
‐ Technology has brought predominantly positive things;
‐ Technological progress is needed, and one has to accept possible disadvantages;
‐ Problems related to technology can be resolved by further developments;
‐ To keep the current life standard, one has to keep up with technological developments, if voluntarily or not.

The participants could state their opinion on a six-point Likert scale (6, strongly agree; 1, strongly disagree).

The obvious differences in the opinions of male and female participants are shown in Figure 12. Due to sample characteristics, a series of non-parametric significance tests Mann and Whitney *U* − *test*) were performed, which revealed two significant gender differences in the items “I always had to deal with technology in my life” (*p* = 0.034) and “Complex technology always irritated me” (*p* = 0.011). The other visually salient differences in “I was avoiding technology whenever possible” (*p* = 0.177) and “A profession in the technology sector would not have been adequate for me” (*p* = 0.051) were not significant on a statistical level.

The motivation to participate in the project is another interesting aspect. Every participant (without exception) either had a severe health problem himself or herself or was directly involved in the support or care of people who had severe health-related problems or suffered from chronic diseases. In this case, no gender differences can be observed. This experience obviously influenced their attitude regarding a system that could improve the situation of affected persons. An observable difference was that if participants had experienced health-related problems themselves, they were willing to allow more installations than others. However, this tendency was again influenced by gender. Women negotiated the installation of each device. These differences in the motivational structure of the participants resulted in a clearly different number of installed devices ranging from 14 down to only two. However, it was interesting that even in the household where only a few devices could be installed, there was a reasonably high accuracy in the recognition of daily activities and deviations. This seems to be due to the fact that the inhabitants had a strict daily routine and the devices were optimally placed.

Regarding their attitude to life and life satisfaction, the participants have a positive overall attitude. However, there is an observable tendency that women are more worried about the future than men. The details would be outside of the scope of this paper; therefore, only the aspects related to technology are presented. Women significantly agree more with the statements “In my life, there are many things that scare me” (*U* -test, *p* = 0.035) and “There are many things that make me sad” (*p* = 0.041). Not surprisingly, women specifically expressed fears related to things to be expected, such as the disturbance of privacy, permanent surveillance or electronic smog in the starting phase of the project. However, those fears more or less disappeared in the course of the project. This can be partly explained as an effect of the unobtrusive installation of the components, which allowed the participants to become increasingly less conscious of the presence of the new technology installed in their homes. In one case, the monitoring changed the attitude of a female participant in an unexpected direction. The first series of motion sensors provided a feedback by default, which was a short flashing, when a motion was recognized. The woman was observing this feedback and felt reassured, specifically when she had to pass the stairs to get to another floor. The feedback told her that the system has recognized her arrival on the other side of the stairs and that in case of a deviation, her trusted person would react. However, most of the other participants found the flashing annoying, and therefore, it was turned off. Although the feeling of being surveilled was reduced in the course of the project, the concerns about potential misuse of data remained. For example, at the start of the project, participants expressed the fear (nourished by a heated public debate on smart metering) that burglars could hack into the system to find out whether or not someone is at home. This fear did not disappear, but it was overcome by the trust the participants had in the university and the Casa Vecchia team.

Socio-psychological findings of interest were also identified on the side of the trusted persons. Specifically, unrelated trusted persons had concerns and even fears regarding their role. They did not want to assume an increased responsibility when agreeing to take part in Casa Vecchia. The impact that a technical system could have on such social aspects could only be partly researched within the project; however, the concerns of the trusted persons are understandable. Without a system providing status information about a known person, the responsibility for this person is evenly distributed over the whole social network (the relatives, the neighborhood, *etc*.). With a system like the Casa Vecchia smartphone, a high proportion or even the whole responsibility is given to one trusted person. Given the previously-discussed technological problems and the behavioral demands, it is not surprising that people were worried about potential consequences.

In summary, the achieved results support the need for projects, such as Casa Vecchia. While our findings relate to many theoretical and lab-based studies, there are simple truths to be measured in the field that cannot be anticipated elsewhere. Further research based in rural environments and using the real homes of real seniors may reveal even more technical and infrastructural insights that will be needed in order to provide AAL to all of the aging population of Europe.

## 5. Discussion

In this paper, the possibilities of disseminating AAL in rural areas were discussed, both on a theoretical level and on real-world data derived from the Casa Vecchia Project. The goal of Casa Vecchia was to build upon the extensive research from the fields of smart home, ambient intelligence and ambient assisted living and to deploy an empirically-valid and commercially-practicable AAL system that would have real-world value for seniors living in their own rural homes. Although much research has been carried out in the related fields, surprisingly little considers the rural area. It was therefore not surprising in general that we had to face many challenges in our attempts to implement AAL in these areas. Several problems were technological, caused, for example, by the infrastructural disadvantages of rural areas. Other problems were related to the smart home/AAL technology itself, as an effect of missing standards, specifically in the segment of wireless smart home systems. The lack of compatibility and interoperability discussed in Section 2 is a major drawback. This impression was also supported by the electricians we involved in the project. In contrast to urban areas, where electricians are typically specialists in a certain segment or for a specific system and manufacturer, electricians in rural areas have to be generalists. They would therefore be more than happy if there were more standardization and regulation, such as is the case with wiring standards, the characteristics of fuse box components or the connector specifications of standard components. Currently, they, in general, advise their customers against smart home technology, because of the probable problems with incompatibility issues, which obviously contradicts the goals of AAL.

Given these factors, we had to invest a lot of effort into the customization of components, but the resulting platform presented in this paper has, in comparison to other systems on the market, several advantages. One is that it is generic. The components used to integrate appliances, such as the stove, into a smart home system are characterized by simple interfaces, which would also allow their integration into any other smart home system. The other advantage of our system is that we could show how to integrate three different smart home systems, learn about the specifics and try to generalize them. This is an important differentiation and constitutes a concrete example of compatibility and interoperability, which, in the literature, is often only discussed on a theoretical level [47]. Many systems already on the market provide similar functions to those the Casa Vecchia system currently provides. However, because of the open architecture, an integration of additional functions, devices and services is possible. This is also true for other areas, such as the connection infrastructure. Because of the problems with Internet connectivity, we integrated two alternative, wireless (3G) Internet connections, as well as wired ones. This flexibility could solve some of the connection related problems of the starting phases.

What we consider as another essential outcome of Casa Vecchia was that the goal of unobtrusiveness could be reached to a reasonable degree. The whole group of participants responded that they did not feel disturbed by the installation or by the technology itself. As a result, we can consider our approach as a small step towards Mark Weiser's goal that “the most profound technologies are those that disappear”.

That said, there are still a lot of problems to solve, specifically the gender related issues. ICT has still a male touch and obviously does not adequately address the needs of the women, who will be, according to the estimations of the demographic change, the largest target group for AAL technology. A gender sensitive approach, as, for example, discussed by [48], is therefore necessary.

Another area for potential enhancements is interaction (control, programming, configuration) with AAL systems. As illustrated in the example of the pairing mechanisms, current systems do not offer an appropriate level of usability that enables laypersons to easily interact with a system. A combination of automated and interactive functionality could be a solution. For example, cellar lights in some Casa Vecchia households were frequently not switched off by the participants in the past. Because the cellar is, like the stove, another potentially dangerous area in a household (regarding the probability of falls), they were also integrated for security reasons. A simple, automated solution was to to turn off the lights automatically after a certain time delay (in combination with a motion detector showing that there was no activity in the respective room). Alternatively, the participants could get feedback about the status of the lights on the central unit with the possibility to switch them off remotely. The latter requires appropriate means of interaction, which we tried to establish with the alternative interface concepts of informative art and the alternative modes of interaction (e.g., speech).

These results show the potential of the platform, on one hand, and the possibilities to overcome interaction barriers, on the other. One can envision many ways in which the active examination of ICT could be specifically beneficial for inhabitants of rural areas. Many services could be plugged into such systems if an adequate foundation were present. The major goal of Casa Vecchia was to establish and thoroughly evaluate such a foundation. With appropriate technology, inhabitants of rural areas could meet many of the goals of AAL. For example, they could keep in touch with the outside world, which supports the goal of inclusion. This could reduce the drawbacks of long driving distances and age-related decreased mobility. Other services could include the coordination of goods and supplies. A phenomenon that is strongly related to rural escape is the reduction of infrastructure, for example, small grocery stores are rare and many have been replaced by shopping malls or large stores in urban or suburban areas. Of course, it would not make sense for chain stores to deliver goods on an individual basis to remote areas, but if the logistics and orders could be handled in a coordinated way, such a business model could be lucrative. The Casa Vecchia system could provide an appropriate foundation and interfaces.

This and other further developments could contribute to the attractiveness of rural areas as a living environment for those who are already there, but could even motivate others to consider rural areas as a place for a retirement home. This would also require the involvement of support organizations and informal caregivers. The technical system can provide peace of mind [49] to the relatives, friends or other trusted persons and also give confidence to the elderly that if something unexpected occurs, somebody from outside will be informed and can react. However, as described in Section 4, to hand over the whole responsibility to one trusted person would not be optimal, because of the feeling of being overburdened. Professional service providers, such as mobile care organizations, emergency call centers, *etc*., have to be considered, too. The Casa Vecchia platform provides interfaces that enable the integration of these kinds of systems. Beside the possibility of a technical connection, care organizations would also have to reconsider their business models. As discussed above, the motivation to participate in the project was related to the fact that the participants had experiences with health issues in the past. One insight they drew out of this experience was that there is a gap in support services. If people are fully independent, they can stay at home. If there is some probability of an incident (e.g., because of similar episodes in the past or increasing age), a person would have to move to a nursing facility or the person herself and the social network would have to take over an unpredictable risk. The Casa Vecchia participants shared the opinion that the services that are offered are too inflexible, too costly and otherwise inappropriate and, therefore, are not accepted. Technical solutions and services must be further developed hand in hand, in order to meet the needs of the target group.

An important aspect is related to data acquisition and data processing. In Casa Vecchia, this aspect was only partly of relevance. As mentioned, the participants are aware that data can be misused, but on one hand, the benefit of the security features is bigger than the potential dangers, and on the other hand, the level of trust in our institution, the involved persons and the security features were sufficient to overcome the concerns. Services offered on the market will also have to address issues of trust and security.

Because the participants got the installation and the devices for free, the Casa Vecchia Project could not investigate questions related to the willingness of the participants to spend money on ICT supporting AAL. However, a tendency could be observed. Specifically, largely independent seniors (who represent the majority of our sample) do not see an immediate benefit in the purchase of state-of-the-art technology. In contrast to taking out an insurance policy (even for very improbable events beyond our control), the investment in ICT to enhance security, safety and comfort does not seem to be a sufficient motivation.

## 6. Conclusions

Many questions are still unanswered or could not be addressed in the Casa Vecchia project, because of its scope and the original goal of being an initial feasibility study. The major result is that it is not possible to thoroughly research AAL without going into the field. Although the basic technology has reached a level of maturity that can be considered as adequate to provide the required features, it could be pointed out that there is still potential for improvement, even on the technical level. A company developing and producing electronic components could, in our opinion, quite easily integrate our four-component device into only one component, if they were aware of the demand. The results that were achieved in relation to usability issues were expected; others were not. Making AAL a successful approach for the future therefore requires a move away from the conventional research approaches currently dominating the field of AAL. Theoretical models and artificial simulations should be replaced by more phenomenological approaches that stem from a long history in sociology, anthropology and psychology.

AAL could specifically be of great benefit to inhabitants of rural areas, but the systems have to meet certain requirements to fulfil the needs and be accepted by their users. On the basis of an adequate technology, the challenges of the demographic change could be faced in a better way. Although not statistically representative, the feedback of the participants in Casa Vecchia is worth noting. They stated unanimously that their perspective on the future became more positive because of the project. More specifically, they stated that the adaptations of the technology to suit their needs and other in-project solutions gave them the impression that they could stay at home in their old age. Relatives who feel responsible for the previous generation have a higher peace of mind when given the option of being informed when a problem occurs without having to disturb the elderly person with frequent “test-calls”. Finally, local enterprises (in our case, the electricians) can strengthen their role and stand out from competitors because of enhanced knowledge, on the one hand, and the vicinity and possibility to provide fast support, on the other.

## Figures and Tables

**Table 1.** Overview on the demographic characteristics of the Casa Vecchia sample.

**Age**	**Gender**	**Persons in Household**	**Marital Status**	**Former Profession**	**Trusted Person**
73	m	1	widowed	worker, farmer	son
62	f	2	married	hairdresser, housewife	son
64	f	2	married	nurse, office clerk	daughter
64	f	2	married	translator	partner
66	m	2	partnership	social insurance clerk	son
73	f	1	widowed	teacher	son
64	m	2	married	kindergarden nurse	neighbor
50	f	2	married	branch manager, clothing	partner
71	m	2	married	mechanical engineer	son
63	f	2	married	primary school teacher	daughter in law
69	m	2	married	company owner, consultant	son
66	f	1	divorced	nurse	daugther
60	m	2	married	company car driver	neighbor
61	f	2	married	hospital manager	neighbor
70	f	1	widowed	hospitality industry	son
71	f	1	widowed	owner of transport company	sister
62	f	1	widowed	school janitor	daughter
64	m	2	married	highschool teacher	sister
67	f	1	widowed	office clerk	daugther, grandson
69	f	1	divorced	support for crime victims	daugther
70	m	2	married	M.D.	partner
83	f	1	widowed	office clerk	daugther

				**Mean Age**	66.45
				**Age Standard Deviation**	6.39
				**Main Persons in Project**	
				**Female:**	14
				**Male:**	8
				**Sample size Main Persons:**	22
				**Sample size with Partners**	35
				**(without Trusted Persons)**	

**Table 2.** Combination and number of devices installed in the participating households.

**Household**	**Inhabitants**	**No. of Devices**	**Device Type and No.**			
HH 2	1	8	MS3, DWC1, PUI1, SA1, DS2
HH 11	1	12	MS2, DWC2, DS2, S1, AOB, SA4
HH 13	2	4	DWC1, AOB, SA2
HH 14	1	7	MS2, DS1, SA2, AOB, SD1
HH 15	1	2	MS2
HH 16	1	4	MS1, AOB, DWC1, SA1
HH 17	1	8	MS2, AOB, DWC2, DS1, SA2
HH 18	2	14	MS6, SA5, DS1, DWC1, AOB	**Legend**		
HH 19	2	7	MS3, DWC2, AOB, DS1	**Abbr.**	**Long Name**	**Sum**

HH 20	2	13	MS5, DWC2, AOB, SA3, SD2	MS	Motion Sensor	**47**
HH 21	2	7	MS1, AOB, DS1, SA4	SA	Switchable Actor	**35**
HH 22	2	6	MS1, DWC1, AOB, DS2, SA1	DWC	Door Window Contact	**26**
HH 23	2	8	MS2, DWC3, DS1, SA1, AOB	AOB	All off Button	**17**
HH 24	2	8	DWC1, AOB, SA3, S1, RC1, DS2	DS	Device Sensor	**16**
HH 27	2	6	MS1, AOB, SA3, DWC1	SD	Smoke Detector	**3**
HH 29	2	4	MS2, AOB, DWC1	S	Generic Switch	**2**
HH 30	2	6	MS4, DWC2	PUI	Push Button Interface	**1**
HH 32	1	7	MS3, DWC1, AOB, DS1, SA1	RC	Remote Control	**1**

HH 33	1	6	MS2, DWC1, SA1, AOB			
HH 34	2	3	MS2, DS1,			
HH 35	2	4	MS2, DWC2			
HH 36	1	4	MS1, DWC1, AOB, SA1			

	**Sum**	**148**				
	**Mean**	**6.7**				
	**Max**	**14**				
	**Min**	**2**	

**Table 3.** Devices and their (typical) association with daily time slots.

**Daytime:**	**Getting up**	**Morning**	**Noon**	**Afternoon**	**Evening**	**Night**		
HH 2	MS	MS, SA, DWC	SA	MS, DWC	DS	MS
HH 11	MS	MS, DWC, DS,AOB	SA, DS	MS, DWC,AOB	DS,SA	MS
HH 13	DWC	DWC, AOB	DWC, SA	DWC, AOB	DWC	AOB, SA
HH 14	SA, DS	MS	SA, DS	MS, SA, DS	DS	MS
HH 15	MS	MS	MS	MS	MS	MS
HH 16	DWC, SA	MS, AOB	DWC,SA	MS, AOB	DWC, SA	MS, DWC
HH 17	DS, SA	MS, AOB	SA, DS	MS, AOB	SA, DS, AOB	MS
HH 18	DS, SA	MS, DWC	DS, SA	MS, AOB, DWC	MS, DWC	MS, DWC	**Legend**	
HH 19	DS	MS, AOB, DWC	DS, DWC	MS, DWC, AOB	DS, MS	MS	**Abbr.**	**Long Name**

HH 20	SA, DS	MS, DWC, AOB	SA, DS	MS, DWC, AOB	SA, DS, DWC	MS, SA, DS	MS	Motion Sensor
HH 21	SA, DS	MS, DS, AOB	SA, DS	MS, SA, AOB	MS, DS	MS, SA	SA	Switchable Actor
HH 22	MS	MS, DWC, AOB	SA, DS	MS, DWC, AOB	DS, SA	MS, DWC	DWC	Door Window Contact
HH 23	SA, DS	MS, DWC, AOB	SA, DS	MS, DWC, AOB	DS, SA	MS, DWC	AOB	All off Button
HH 24	SA, DS	SA, DS, DWC, AOB	SA, DS, RC,	SA, DS, AOB	DS, SA, RC	AOB, DWC	DS	Device Sensor
HH 27	SA	MS, AOB, DWC	SA, DS	SA, AOB, DWC	SA, DS	MS, DWC, AOB	SD	Smoke Detector
HH 29	MS, DWC	DWC, AOB	MS, DWC	DWC, AOB	MS	MS	S	Generic Switch
HH 30	MS	MS, DWC	MS	DWC, MS	MS	MS	PUI	Push Button Interface
HH 32	DS, SA	MS, DWC, AOB	DS, SA	MS, DWC, AOB	DS, SA	MS, DWC	RC	Remote Control

HH 33	SA, DWC	MS, AOB	SA, DWC	MS, AOB	SA, DWC	MS		
HH 34	DS	MS	DS	MS	DS, MS	MS		
HH 35	MS	DWC, MS	MS, DWC	MS	MS, DWC	MS		
HH 36	SA, DWC	MS, AOB	SA, DWC	MS, DWC, AOB	MS, DWC	MS		

**Table 4.** Probability of events on the example of the weekday, Monday.

**Day: Monday Hours (AM)**	**P**(*a*(*j, k*))	**Hours (PM)**	**P**(*a*(*j, k*))
0	0.04	12	0.94
1	0.00	13	0.57
2	0.00	14	0.86
3	0.04	15	1.00
4	0.06	16	0.96
5	0.23	17	0.98
6	0.90	18	0.76
7	1.00	19	0.75
8	0.98	20	0.56
9	0.88	21	0.90
10	0.98	22	0.77
11	0.94	23	0.37

**Table 5.** The number of collected events per participating household.

**Household**	**No. of Devices**	**Setup Date**	**Days Online**	**Events**
HH Test 2	4	18.01.2011 15:43	1137	127017
HH 11	12	26.04.2011 15:31	1039	47408
HH 13	4	28.04.2011 11:23	1037	107823
HH 14	7	10.08.2011 17:46	933	99937
HH 15	2	21.04.2011 12:20	1044	114482
HH 16	4	21.04.2011 18:37	1044	317075
HH 17	8	03.05.2011 15:07	1032	55030
HH 18	14	01.06.2011 13:39	1003	355219
HH 19	7	16.05.2011 19:20	1019	50372
HH 20	13	03.08.2011 10:43	940	201158
HH 21	7	30.05.2011 15:12	1005	260104
HH 22	6	30.05.2011 12:06	1005	141790
HH 23	8	06.06.2011 15:49	998	111643
HH 24	8	30.06.2011 11:15	974	137542
HH 27	6	15.06.2011 19:43	989	25289
HH 29	4	25.07.2011 15:32	949	3129
HH 30	6	15.12.2011 11:50	806	29034
HH 31	6	14.05.2011 13:32	1021	32426
HH 32	7	09.08.2011 16:41	934	52996
HH 33	6	15.12.2011 14:47	806	34603
HH 34	3	08.02.2012 14:15	751	28939
HH 35	4	09.02.2012 13:57	750	22402
HH 36	4	03.05.2012 12:28	666	74485

		**Days**	**Years**	**Events**
	**Mean**	951.39	2.61	105647.96
	**Standard Deviation**	224.86	0.33	93648.42
	**Maximum**	1137	3.12	355219
	**Minmum**	666	1.82	3129
	**Sum**	21882	59.95	2429903

**Table 6.** Usage frequency of the features accessible over the informative art GUI on the central unit.

**Feature**	**Frequency of Use**	**No. of households using the feature**
News	1424	11
Doorcam	1199	2
Weather	405	14
Mail	205	3
